# In-situ electrochemical analysis of microbial activity

**DOI:** 10.1186/s13568-018-0692-2

**Published:** 2018-10-04

**Authors:** Ariane L. Martin, Pongsarun Satjaritanun, Sirivatch Shimpalee, Blake A. Devivo, John Weidner, Scott Greenway, J. Michael Henson, Charles E. Turick

**Affiliations:** 1 Department of Biological Sciences, Life Sciences Facility, Clemson University, Clemson, SC USA; 2 Department of Chemical Engineering and Computing, University of South Carolina, 541 Main Street, Columbia, Columbia, SC USA; 3 Savannah River National Laboratory, Environmental Science and Biotechnology, Aiken, SC USA; 4 Savannah River Consulting, 301 Gateway Drive, Aiken, SC USA

**Keywords:** Bioprocess, Fermentation, In-situ monitoring, Cyclic voltammetry, Electrochemical impedance spectroscopy, Charge transfer resistance

## Abstract

Microbes have a wide range of metabolic capabilities available that makes them industrially useful organisms. Monitoring these metabolic processes is a crucial component in efficient industrial application. Unfortunately, monitoring these metabolic processes can often be invasive and time consuming and expensive, especially within an anaerobic environment. Electrochemical techniques, such as cyclic voltammetry (CV) and electrochemical impedance spectroscopy (EIS) offer a non-invasive approach to monitor microbial activity and growth. EIS and CV were used to monitor *Clostridium phytofermentans*, an anaerobic and endospore-forming bacterium. *C. phytofermentans* ferments a wide range of sugars into hydrogen, acetate, and ethanol as fermentation by-products. For this study, both traditional microbiological and electrochemical techniques were used to monitor the growth of *C. phytofermentans* and the formation of fermentation products. An irreversible reduction peak was observed using CV beginning at mid-logarithmic phase of growth. This peak was associated with *C. phytofermentans* and not the spent medium and was indicative of a decrease in carbon and energy sources to the cells. Additionally, EIS analysis during growth provided information related to increased charge transfer resistance of the culture also as a function of carbon and energy source depletion. Results demonstrate that CV and EIS are useful tools in the monitoring the physiological status of bioprocesses.

**Electronic supplementary material:**

The online version of this article (10.1186/s13568-018-0692-2) contains supplementary material, which is available to authorized users.

## Introduction

A variety of industrial processes rely on the astounding diversity of microbial metabolic capabilities as well as recent technological advances, all leading to an increase in bioprocess technologies in industries such as food and beverage, pharmaceutical manufacturing, and energy production (Demain 2000; Vojinović et al. 2006). Monitoring the growth and activity of the various microbes involved is an essential component of bioprocesses for process efficiency. Even small changes in the growth environment, such as pH, temperature and pressure can affect metabolic processes carried out by the microbes (Vojinović et al. 2006; Skibsted et al. 2001). Thus, there is a need for rapid and active analysis methods for monitoring growth and activity of microbes (Skibsted et al. 2001; Hobson et al. 1996; Alves-Rausch et al. 2014).

Costs for off-line monitoring including sample collection and analysis necessitate a somewhat minimalistic approach for data collection which decreases the opportunity for early problem detection (Vaidyanathan et al. 2001, Schügerl 2001; Marose et al. 1998). Although, real-time monitoring with the use of on-line sensors is ideal, monitoring gas phase parameters has not provided sufficient analytical results, mostly due to problems associated with liquid-to-gas mass transfer (Pauss et al. 1990). On-line monitoring with in situ electrodes also has problems related to electrode biofouling and therefore requires sufficient maintenance, with associated time and cost for reliable data (Harms et al. 2002).

Monitoring and sampling should be conducted to not contaminate or negatively affect the culture (Vojinović et al. 2006; Harms et al. 2002; Clementschitsch and Bayer 2006; Beutel and Henkel 2011). Additionally, the technology used to monitor the bioprocess must be able to survive harsh conditions, such as sterilization and pressure changes (Harms et al. 2002; Clementschitsch and Bayer 2006; Beutel and Henkel 2011).

Many of the in situ monitoring technologies presently being used often have issues of measurement drift because of precipitation of biological materials, changes in medium composition and the growth of the organisms, which can result in thick biofilms on electrodes (Harms et al. 2002; Clementschitsch and Bayer 2006). Drift results in inaccurate measurements and/or the need for frequent recalibration (Harms et al. 2002). Critical research in this field relates to measurement technology for biomass and product concentration as well as the metabolic state of the cultures (Landgrebe et al. 2010). For example, under batch fermentation conditions, knowledge of growth cycle completion will result in more efficient time management and therefore cost savings for the industry concerned. Additionally, an indication of appropriate sampling time is also needed for efficient bioprocessing. In summary, in situ monitoring is required to improve the efficiency of bioprocesses. The purpose of this work therefore, is to evaluate the use of electrochemical methods for on-line bioprocess monitoring.

Electrochemistry provides a noninvasive approach to monitor microbial activity and allows for the monitoring of electron flow within a microbial community as a function of carbon and energy source utilization. Advantages include real-time or near real-time results with little to no sample collection. This method of monitoring is possible because microbial cells are composed of charged components. For example, the microbial cell surface is negatively charged resulting from constituents such as membrane glycoproteins (Markx and Davey 1999). This charge attracts positive ions to the microbial cell membrane forming a double layer (Markx and Davey 1999). Moreover, the lipid bilayer of the cell and the ions contained within the cytoplasm allow the cell to interact electrochemically (Markx and Davey 1999). For this interaction to take place a potential must be applied to the electrical system.

Cyclic voltammetry (CV) measures redox active components in the system [Bard and Faulkner (1980)], and thus, the electrochemical activities of bacteria present (Rabaey et al. 2004; Fricke et al. 2008). In cyclic voltammetry, the current is measured as a range of potentials is applied (Harnisch and Freguia 2012; Ronkainen et al. 2010). CV was shown to be useful in monitoring biofilms throughout the early stages of development on electrodes and provides utility in monitoring biofouling associated with industrial operations (Vieira et al. 2003). Changes in peak currents during CV evaluation of microbial cultures are related to cell viability and metabolic status (Ci et al. 1997; Matsunaga and Namba 1984). In addition, rapid voltammetric scans also remove biofilms from electrodes (Vieira et al. 2003) indicating that this technique can be used to clean electrodes in situ. Consequently, CV has the potential to monitor cellular activity in situ for extended periods without interference from biofouling.

Electrochemical impedance spectroscopy (EIS) is used to understand many phenomena in electrochemical systems to provide information related to the bulk phase as well as inner and outer interfaces of materials. EIS measures the impedance by imposing low amplitude perturbations of the current or voltage over a wide frequency range. Plots of the components of impedance in the complex plane can then be analyzed to assess the processes affecting the resistance of the system and to estimate the time constant for these processes. Analyses of these spectra have used equivalent circuit models that qualitatively relate physical parameters to phenomena occurring in electrochemical cells. The EIS of biological systems reveals changes in electrical properties with frequency (Zhbanov and Yang 2017). At 100 kHz and lower, signals are associated with lipid membranes, counter ion diffusion, cellular membrane potential, and displacement of charged ions surrounding charged membranes (Zhbanov and Yang 2017; Heilman et al. 2013).

In this study, we used the electrochemical techniques (CV and EIS) during the growth of *C. phytofermentans.* We hypothesized there would be changes in the electrochemistry that can aid in the detection of growth and physiological status of the C. *phytofermentans*. These techniques offer the advantage of monitoring the physiological status of the cells during growth and bioprocess conditions being monitored.

## Materials and methods

### Strain and growth conditions

*Clostridium phytofermentans* (ATCC 700394) was chosen as the bacterial species for this project to evaluate the use of electrochemistry as a possible method to measure bioprocess reactions. *C. phytofermentans* is a rod shaped (0.5–0.8 × 3–15 µm), endospore-forming obligate anaerobe. Endospores are round and terminal, usually 0.9–1.5 µm. *C. phytofermentans* is motile with one or two flagella present and has been shown to grow on a large variety of organic substrates including those found in plant biomass, such as cellulose (Warnick et al. 2002). Fermentation end products include carbon dioxide, hydrogen, acetate, and ethanol (Warnick et al. 2002; Jin et al., 2011; Olson et al. 2012). *C. phytofermentans* was first described in 2002, after being enriched from forest soil as a novel cellulolytic species (Warnick et al. 2002). Its genome was later shown to contain the highest number of cellulases and hemicellulases of any sequenced clostridial genome (Olson et al. 2012; Jin et al. 2012). Although not yet used at the industrial level, advances in genetic tools have the ability to increase its cellulytic and hemicellulytic potential making it a possible candidate for large-scale bioprocesses (Olson et al. 2012).

*C. phytofermentans* was grown in GS-2C medium, which was prepared as follows per liter distilled water: 6.0 g yeast extract, 2.1 g urea, 2.9 g K_2_HPO_4_, 1.5 g KH_2_PO_4_, 10.0 g 3-(*N*-morpholino) propanesulfonic acid (MOPS), 3.0 g trisodium citrate dehydrate, and 2.0 g l-cysteine HCl (Warnick et al. 2002). Final pH was adjusted to 7 with 5 N NaOH. Following the Hungate method (Miller and Wolin 1974) media were brought to a boil and sparged using high purity N_2_. After cooling, the medium was aliquoted into 125 mL Wheaton serum bottles previously degassed with high purity nitrogen, each sealed with a black butyl rubber stopper (Geo-Microbial Technologies, Inc.) and crimped with an aluminum seal (Wheaton). The butyl rubber stopper had a screen printed electrode inserted through it (Pine Instrumentation #RRPE1001C). Prior to performing experiments, the sealed serum bottle was evaluated for gas tightness by submerging under water. Additionally, serum bottles were kept upside down over night to evaluate for leakage. Aliquots were autoclaved at 121 °C for 15 min. Once cooled, the aliquot was amended with a filter sterilized, deoxygenated cellobiose solution to a final concentration of 1 g L^−1^. A 2% inoculum of a stationary phase (sporulated) culture of *C. phytofermentans* was aseptically added using anoxic techniques.

### Cell density measurements

Liquid samples (1 mL) were collected during incubation and then placed into a disposable plastic cuvette for optical density measurements at 550 nm using a UV/Vis spectrophotometer (Shimadzu UV-2401PC). Liquid samples were stored in a 1.5 mL microcentrifuge tube at 4 °C for subsequent analysis of cellobiose, ethanol, acetate and protein concentration.

### Cell counts

Cell counts were performed using DAPI (4′,6-diamidino-2-phenylindole) staining. To do this, optical density was measured on a 1 ml sample. Of this sample, 900 µL was saved and 100 µL of 20% paraformaldehyde (final concentration, 2%) was added to preserve the sample for up to 48 h at 4 °C. After at least 20 min (but no more than 48 h), dilutions were made using sterile sodium buffered saline (pH 7.2). Cells were filtered and stained for 5 min with 2 µg mL^−1^ DAPI. Imaging was conducted using an epifluorescence microscope (Nikon Eclipse E600) with at least five fields per filter (GVS Life Sciences, poretics polycarbonate track etched black 25 mm 0.2 µm) counted with the aid of ImageJ software.

### Hydrogen production

Hydrogen production was measured using gas chromatography. Headspace samples were collected at the previously stated times. Headspace pressure was measured using a digital manometer (Dwyer Series 477). Headspace samples were collected using a gastight 250 µL glass syringe. The samples were injected into a gas chromatograph (Agilent 7890A) with a HP Plot Molesieve column (Agilent J&W, 30 m, 0.32 mm, 25 µm). The following operating conditions were used: inlet at 105 °C splitless, 5.75 psi, total flow 3.5 mL/min with the oven temperature held at 85 °C for 10 min. The detector was a thermal conductivity detector set at 275 °C with a reference flow at 17.0 mL/min and makeup flow at 5.0 mL/min. Argon gas was used as the carrier gas at a flow rate of 0.4 mL min^−1^. Peak analysis was performed using Agilent Chemstation, Enhanced Data Analysis G1701DA ver D.00.00.38 and Microsoft Excel.

### Cellobiose utilization, ethanol and acetate production

High performance liquid chromatography (HPLC) analysis using the supernatant collected during the preparation of the stored liquid samples for protein analysis was performed to measure acetate and ethanol concentration as well as cellobiose utilization. To do this, 500 µL of the supernatant was placed in a 2.0 mL screw-top glass vial and capped with a septum. Samples were analyzed using HPLC (Agilent 1200 series) equipped with a refractive index detector with an Aminex HPX-87H column (Bio-Rad #125-0140) with the following specifications: 300 × 7.8 mm, 9 µm particle size with an attached Cation H micro-guard, 30 × 4.6 mm (Biorad, # 125-0129) column. The samples were run using 5 mM H_2_SO_4_, as the mobile phase with a flow rate of 0.6 mL min^−1^, and a sample volume of 5 µL. Standards of cellobiose, acetate, and ethanol were also run. A standard curve was created and concentrations were determined using the Agilent Chemstation software.

### Cyclic voltammetry

All electrochemical studies were performed on a four-channel potentiostat, VersaSTAT MC (Princeton Applied Research) with data recorded via VersaStudio software version 2.42.3.

**Fig. 1:**
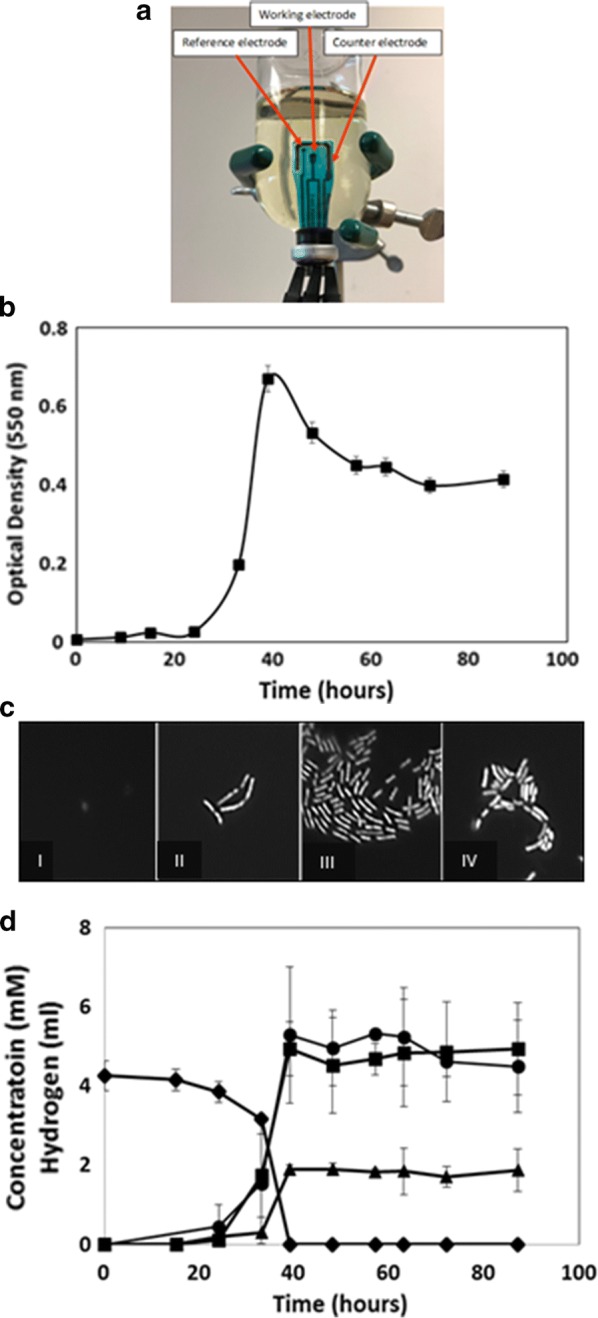
Experimental arrangement and microbiological data. **a** Inverted 125 mL serum bottle with electrode assembly inserted through a black butyl stopper and sealed gas tight. Sterile anaerobic growth medium was inoculated with *C. phytofermentans* spores prior to growth studies. **b** Growth curve of *C. phytofermentans.* Concentration of *C. phytofermentans* is shown over an 87-hour time period. Values are the mean ± S.D. of two duplicate growth studies. **c** DAPI staining of *C. phytofermentans*. DAPI stained images at inoculation (I), lag (II), log (III), and stationary (IV) phases of growth. **d** Conversion of cellobiose (diamond) to acetate (square), ethanol (triangle), and hydrogen (circle) by *C. phytofermentans* during growth. Values are the mean ± S.D. of two duplicate growth studies

Flat patterned electrodes (Pine Instruments) consisted of a 2 mm diameter graphite working electrode surrounded on 3 sides by a graphite counter electrode with a Ag/AgCl reference electrode. Electrodes were fitted through preformed slits in butyl rubber stoppers and sealed gas tight with silicone glue and secured onto serum vials with aluminum seals (Fig. 1). All vials with electrodes were autoclaved *en bloc* prior to studies.

Cyclic voltammetry (CV) was used to monitor changes in the electrochemistry of the system during the growth of *C. phytofermentans* and uninoculated medium. Initial studies involved analyses versus open circuit potential and established variation in reduction peaks relative to growth conditions. Follow on studies involved the same methodology but versus the Ag/AgCl reference.

Additionally, an inoculated control lacking the addition of cellobiose was also tested. Throughout the studies, the flat patterned graphite electrode was first electrochemically cleaned to remove any fouling on the electrode by sweeping the potential rapidly from − 2.0 to + 2.0 V at 1000 mV/s for electrochemical cleaning with three cleaning scans before each analysis.

### Peak characterization

To determine if the observed peak(s) from the CV data were associated with the cell surface or the spent medium bulk phase, two independent approaches were used. First the CV scan rates were varied using the following scan rates: 10, 25, 50, 100, 500, and 1000 mV/s. Peak current was measured using the VersaStudio software and plotted versus scan rate in Microsoft Excel. For this analysis, a linear correlation of peak current to scan rate indicates that the peak is associated with the cell surface whereas a logarithmic fit indicates the peak is associated with the spent medium (bulk phase) (Laviron 1983).

The second approach involved the physical separation of the cells from the spent medium. *C. phytofermentans* was grown to the completion of logarithmic growth during which time a CV peak was observed. At the time at which this peak occurred, 10 mL of culture was anaerobically collected using a sterile needle and syringe and injected into a 30 mL sterile syringe and passed through a 0.22 µm filter into a sterile Balch tube filled with nitrogen gas. CV was conducted on the cell free medium with a 20 mL sterile Balch tube purged with 101 kPa anaerobic N_2_. Additionally, sterile medium was measured to ensure the peak was not associated with medium components.

### EIS methods and modeling

EIS measurements were carried out in sterile and inoculated media with a sinusoidal signal perturbation of 50 mV, with a frequency range of 10^5^ Hz to 10^−2^ Hz. Ten measurements were recorded per decade of frequency with a measurement delay of 2 s. Data were analyzed with ZView software (Scribner Associates). EIS was performed immediately following 3 cycles of voltammetric stripping and conditioning of the electrode (− 2 V to + 2 V: 1000 mV/s). Data were evaluated as Nyquist and Cole–Cole plots. The Nyquist plot represents impedance at each frequency, Z (ω), in complex plan where real (Z′) and imaginary (Z″) impedance are measured. The complex dielectric function (Cole and Cole 1941, 1942) was presented as Cole–Cole plots where real (εʹ) and imaginary (εʺ) permittivity are a function of impedance as defined follows:

1 $$ \varepsilon ^{\prime} = \frac{ - Z ^{\prime \prime} }{{(Z ^{\prime^{2}} + Z ^{\prime \prime^{2}} )\omega C_{0} }} $$

2 $$ \varepsilon ^{\prime \prime} = \frac{ - Z ^{\prime }}{{(Z ^{\prime 2} + Z ^{\prime \prime 2} )\omega C_{0} }} $$

where ω = angular frequency and *C*_0_ = capacitance of an empty cell.

### Circuit model and description of parameters

Impedance and permittivity data from EIS analyses were interpreted using circuit models to determine electrochemical parameters relevant to microbial activity throughout the growth cycle. For circuit model development the elements are sized using a Complex Non-linear Squares (CNLS) method to give the best fit of the equivalent circuit model to the experimental data. Typical electric circuits used to model impedance data are resistor (zero frequency impedance, R  ), capacitor (C  ), constant phase element (CPE  ), and Warburg impedance (W  ). The relationship between the impedance and equivalent elements are shown below.

3 $$ {\text{Z}}_{\text{R}} = {\text{ R}} $$

4 $$ {\text{Z}}_{\text{C}} = { 1}/(j\omega {\text{C}}):j{\text{is imaginary number }}(\surd - 1) $$

5 $$ {\text{Z}}_{\text{CPE}} = { 1}/(j\omega )^{\text{p}} {\text{T}}:{\text{ p }} = {\text{ phase angle}},{\text{ T }} = {\text{ capacitance}} $$

6 $$ {\text{Z}}_{\text{w}} = { 1}/{\text{T}}\surd (j\omega ) $$

## Results

### Microbiological analysis of *C. phytofermentans* growth

*Clostridium phytofermentans* had approximately a 3-day growth cycle with a calculated doubling time of 2.3 h per generation. Cell counts indicated that lag phase of growth occurred on day one, (which would have included endospore germination), logarithmic phase of growth on day two and stationary phase of growth on day three (Fig. 1). Chromatography results corroborated this with cellobiose utilization as well as acetate and ethanol production beginning with log phase growth initiating at about 24 h (Fig. 1). The remaining cellobiose was utilized on the second day. Acetate, ethanol, and hydrogen production also peaked on the second day and remained constant throughout.

### CV results and data analysis

**Fig. 2:**
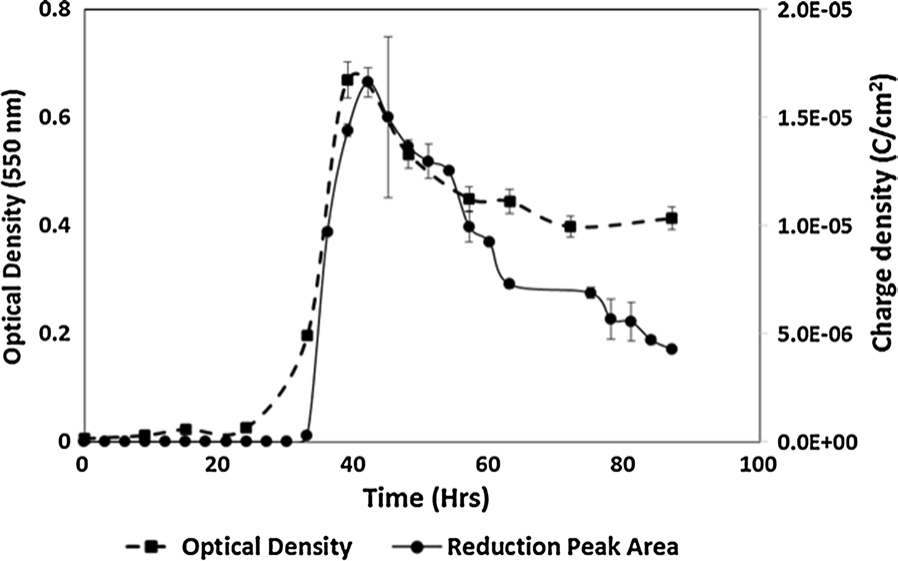
Cell density correlated with reduction peak charge density. Variations in charge density of the reduction peaks generally followed growth data from mid-log phase growth until stationary phase

During mid-log phase growth (~ 36 h) an irreversible reduction peak was detected (relative to abiotic controls) with the largest peak current as log phase growth transitioned to stationary phase (42 h) and then continued to diminish into stationary phase (87 h) corresponding somewhat with cell concentration over time (Fig. 2 and Additional file 1: Fig. S1).

**Fig. 3:**
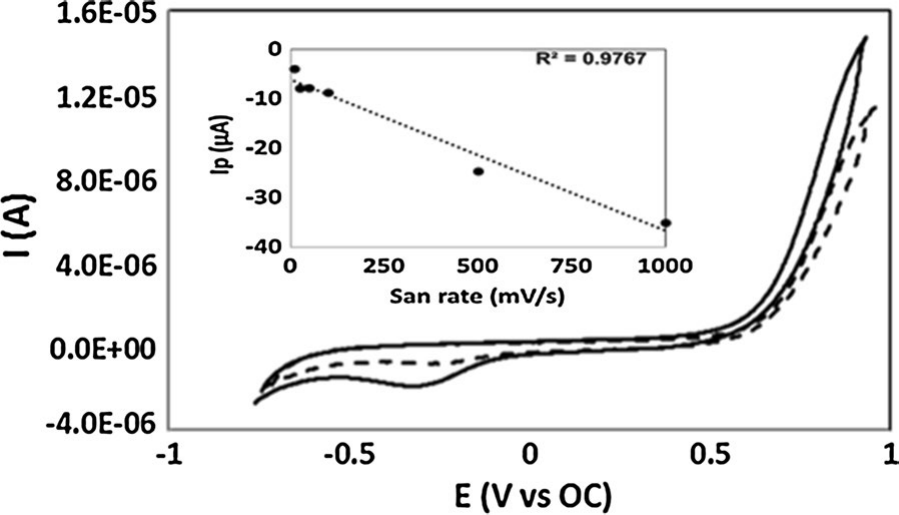
Reduction peak confirmed to be associated with *C. phytofermentans*. With bacterial cells (solid line) in the medium, a reduction peak is observed. When the bacterial cells are removed (dashed line), the reduction peak is no longer observed. Inset: Linear regression of peak current versus scan rates. The peak current of each scan rate was plotted and corresponded surface associated electron transfer based on linear regression analyses

The nature of the reduction peak was evaluated by removing the bacterial cells from the medium and evaluating only the spent medium by CV. As shown in Fig. 3, a reduction peak is not observed in the medium after cells were removed but only in the presence of bacteria. In a separate complimentary study, peak current of the voltammograms was plotted against various scan rates demonstrating a linear fit and thereby corresponding to electron transfer at the cell surface but not the bulk phase (Fig. 3 inset).

### EIS results and data evaluation

**Fig. 4:**
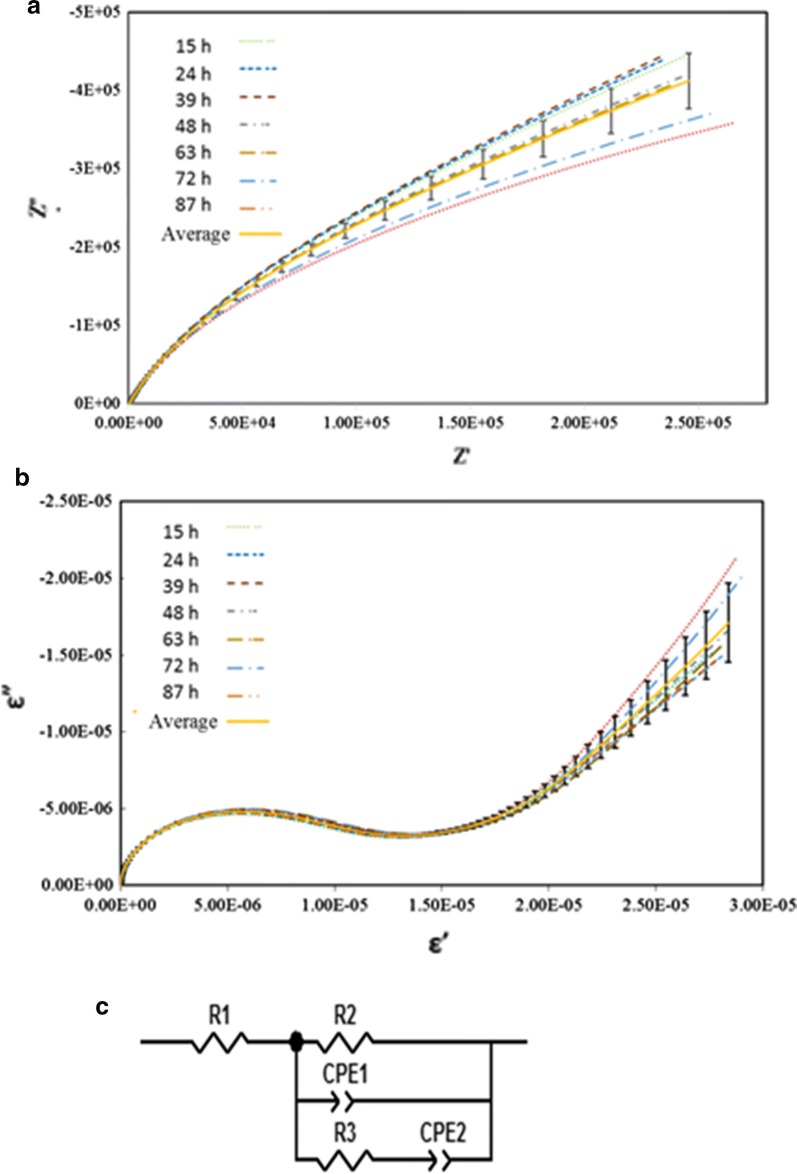
EIS results from abiotic controls from both Bode (**a**) and Nyqyist (**b**) plots demonstrated minimal drift and good precision from 100 kHz to 0.01 Hz. throughout the course of the study. The equivalent circuit (**c**) used to fit the data where R1 represents electrolyte resistance at high frequency, CPE1 and R2 represent a constant phase element and charge transfer resistance, respectively at lower to medium frequencies, with CPE2 and R3 representing a constant phase element and a modification of the charge transfer resistance respectively at low to high frequencies

Figure 4 shows the predictions of both Nyquist plots and Cole–Cole plots under abiotic control at temperature of 35 °C from initial stage to 87 h. The frequency range was set from 10^−2^ to 10^5^ Hz. Note that these predictions are a good fit when compared to experimental data. Figure 4c presents the equivalent circuit used in this study. It consists of resistor (R1) that represents the electrolyte resistance, connected with parallel circuits of resistor (R2), constant phase element (CPE1) and a series of resistor (R3) and constant phase element (CPE2). With this circuit, R2 and CPE1 represent the value and shape at lower to medium frequency and R3 and CPE2 control the value and profile at low to high frequency. From Fig. 4, the Nyquist plot shifted from top to bottom with time especially at the lower frequency and the Cole–Cole plot shifted from bottom to top also at lower frequencies.

**Fig. 5:**
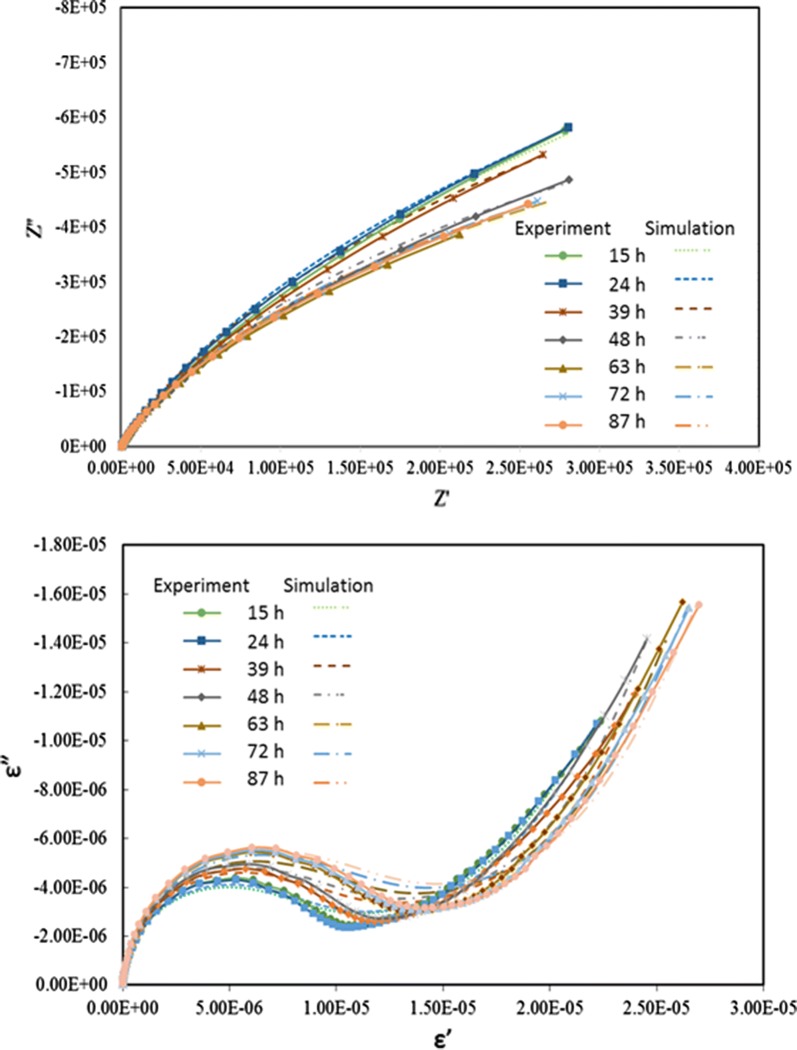
EIS results of cultures of *C. phytofermentans* from both Bode (top) and Nyquist (bottom) plots during growth on cellobiose. The data were fitted to the equivalent circuit (Fig. 4) with good precision

Figure 5 presents the EIS plots for C*. phytofermentans* during growth on cellobiose. These plots include both experimental data and predictions. The shapes of Nyquist plots are similar to the case without cell culture as shown in Fig. 4. However, the graph starts to move up from the initial stage until 20 h then it shifts down toward the end of experiment. For the Cole–Cole plot, the changing pattern of the graph is similar to the Nyquist plot for only medium to low frequency. At the range from high to medium frequency, the Cole–Cole plot moved down from initial stage to 20 h after which it moves up until the end of the run.

**Fig. 6:**
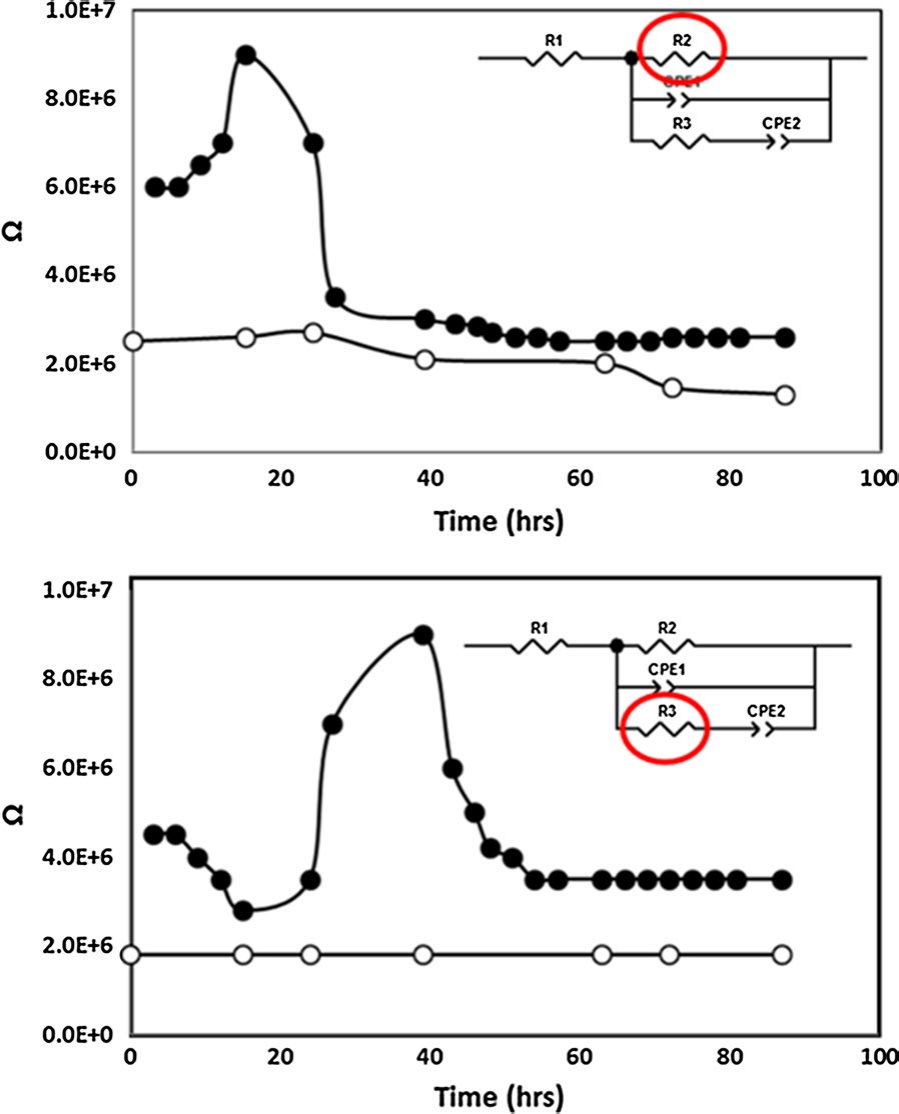
The equivalent circuit used was in good agreement with the experimental data and provided information related to changes in R2 (top), ascribed to ionic flux across bacterial membranes and R3 (bottom), ascribed to metabolic activity and growth of *C. phytofermentans* (black circles) relative to abiotic controls (white circles)

The change of circuit parameters during abiotic control and the growth of *C. phytofermentans* are shown in Fig. 6. This figure provides the profiles of R2 and R3. It clearly shows that R2 has very small decay with time and R3 is unchanged under abiotic control. R1 values (Additional file 1: Fig. S2) for the abiotic controls remained constant through the study while that of the *C. phytofermentans* culture changed little during growth but registered slight changes before growth and shortly after growth ceased. This result could reflect slight changes in fluid conductivity of the culture. CPE 1 and CPE2 values for the culture and CPE2 of the abiotic control remained essentially constant throughout the study while the CPE1 control demonstrated some drift over time (Additional file 1: Fig. S3).

When there is metabolic activity of C*. phytofermentans,* the changes of R2 and R3 are significant. For the first 20 h, R2 increased while R3 decreased. After that, R2 decreased drastically meanwhile the R3 significantly increases. Both reached steady state after 60 h.

## Discussion

Here we evaluated electrochemical methods corresponding to microbiological and biochemical analyses for potential as an in situ monitoring strategy that can be employed for a broad range of bioprocesses for real time growth data on demand. The basis for this technology is that the growth and metabolism of microbes depends on a series of oxidation/reduction reactions (Demain 2000). The movement of electrons via metabolic pathways and electron transport chains as a function of carbon and energy source utilization as well as ionic flux across membranes can be measured by electrochemical techniques.

In this study, *C. phytofermentans* was chosen as an example of a microbial culture with potential in industrial microbiology, specifically the ability to ferment a large range of carbohydrates producing acetate, ethanol and hydrogen. Growth of *C. phytofermentans* with cellobiose was similar to previous studies with an approximate 3-day growth cycle (Warnick et al. 2002).

Production of fermentation products coincided with the 3-day growth pattern observed for *C. phytofermentans*. As cellobiose was utilized, cell density increased along with production of acetate, ethanol and hydrogen, until cellobiose was depleted. Cell density generally followed changes in charge density however it is more likely the charge density more accurately relates to the metabolic status of the cells rather than cell density. In particular, the nature of a reduction peak without its concomitant oxidation peak indicates electron uptake and in this case, utilization by the cells. Direct electron uptake from an electrode has been reported in Clostridia related to bioelectrochemical systems and especially associated with NADH/NAD^+^ pathways (Choi et al. 2014).

In the present study, direct electron transfer from electrode to *C. phytofermentans* was demonstrated with a strong linear correlation of peak current vs scan rates as well as an absence in current production with cell free spent medium. Direct electron uptake from an electrode increased the metabolic reducing power of *C. pasteurinium* (Choi et al. 2014) as is evident in a shift of the NADH/NAD^+^ ratio by increasing intracellular reducing power. Here we show the increase in reduction current from bacterial contact to the electrode in the bacterial suspension began at mid-log phase growth when the carbon and energy source was becoming depleted. During this time, a shift in the NADH/NAD^+^ ratio to be more oxidizing is expected in the culture with the decrease in concentration of the carbon and energy source. This loss of intracellular reducing power may predispose the cells to accept extracellular electrons more readily as evident by the increase in reducing current to the bacteria at mid log phase growth in this study.

EIS data provided a broader view of the system electrochemistry. Our model demonstrated that charge transfer resistance at the lower frequencies (R3) was closely correlated with metabolic activity during growth. This provides evidence that the variation in charge density of reduction peaks throughout growth was linked to the ease in microbial electron transfer during growth, with greater difficulty occurring as carbon and energy sources are becoming depleted. The decrease in charge transfer resistance as the culture transitioned into stationary phase growth likely was due to the decreased energy levels of the cells as well as a return to the spore stage. Previous impedance analyses of bacterial cultures also demonstrated an increase in overall permittivity during growth with a sharp decline during sporulation (Sarrafzadeh et al. 2005).

Bacterial growth or catabolic activity was not detected during the first 24 h after inoculation as the inoculum consisted of a stationary phase culture and based on direct counts the culture medium consisted of endospores. Cole–Cole plots however demonstrated an increase in resistance (R2) during lag phase growth several hours after inoculation and then subsided and remained constant as metabolic activity initiated and cellular growth resumed. These data reflect frequency responses that could be related to chemical and physical changes in the medium and could signify rapid ionic flux in the culture medium associated with endospore germination. The purely biophysical and biochemical reactions that occur during endospore germination and leading up to metabolically functioning vegetative cells likely account for the sudden increase in R2 values just prior to growth and carbon source utilization.

In Clostridia, endospore germination is a dynamic event and consists of actions that are biochemical and biophysical in nature, which include a rapid release of monovalent cations and dipicolinic acid leading to degradation of endospore peptidoglycan and then followed by rehydration and finally resumption of metabolic activity (Moir 2006; Paredes-Sabja et al. 2008; Olguín-Araneda et al. 2015). These data offer evidence that activities related to chemical and physical changes in cultures can be detected and differentiated with EIS and circuit models and demonstrate that electrochemical methods will be able to provide valuable, real time information typically obtained from more traditional methods.

In summary, the results of these studies show that CV and EIS are useful as a real-time measure of bacterial growth and physiological status. These results are significant in that an industrial process can be monitored without the need to collect samples, which can disrupt or even contaminate a process. Incorporation of an electrode configuration into a conventional bioreactor will enable the linking of electrochemical data to process controls which offers a potential method of efficient automation of bioprocess controls. Furthermore, this methodology can potentially provide additional insight into various cellular metabolic processes such as those present in industrial fermentations.

## Additional file

**Additional file 1.** **Figure S1** Voltammograms of *C. phytofermentans* during growth. Reduction peaks were evident in the presence of *C. phytofermentans* during growth with variations in the peak current throughout the growth cycle. Insets demonstrate the part of the growth curve that the CVs represent. **Figure S2** Changes in R1 during growth of *C. phytofermentans *(black circles) relative to abiotic controls (white circles). **Figure S3**. Changes in CPE-1 and CPE-2 during growth of *C. phytofermentans* (black circles) relative to abiotic controls (white circles).

## Abbreviations

CV: cyclic voltammetry
EIS: electrochemical impedance spectroscopy
ATCC: American Type Culture Collection
MOPS: 3-(N-morpholino) propanesulfonic acid
DAPI: 4′,6-diamidino-2-phenylindole
HPLC: high performance liquid chromatography
Z′: real impedance
Z″: imaginary impedance
ε′: real permittivity
ε″: imaginary permittivity
ω: angular frequency
C: capacitor
C_0_: capacitance of an empty cell
CPE: constant phase element
R: resistor
