# Supplementary material for: In-situ electrochemical analysis of microbial activity
Source: AMB Express. 2018 Oct 4;8:162. doi: 10.1186/s13568-018-0692-2 (PMC6172163; doi:10.1186/s13568-018-0692-2)
Supplement: Supplementary file 1 — Additional file 1. Figure S1 Voltammograms of C. phytofermentans during growth. Reduction peaks were evident in the presence of C. phytofermentans during growth with variations in the peak current throughout the growth cycle. Insets demonstrate the part of the growth curve that the CVs represent. Figure S2 Changes in R1 during growth of C. phytofermentans (black circles) relative to abiotic controls (white circles). Figure S3. Changes in CPE-1 and CPE-2 during growth of C. phytofermentans (black circles) relative to abiotic controls (white circles). [file 13568_2018_692_MOESM1_ESM.docx]

**Additional file**

**AMB Express**

**In-situ Electrochemical Analysis of Microbial Activity**

Ariane L. Martin^1^, Pongsarun Satjaritanun^2^, Sirivatch Shimpalee^2^, Blake A. Devivo^2^, John Weidner^2^, Scott Greenway^4^, J. Michael Henson^1^ and Charles E. Turick^3*^

^1^ Department of Biological Sciences, Life Sciences Facility, Clemson University,

Clemson, SC USA

^2^ Department of Chemical Engineering and Computing, 541 Main Street
Columbia, University of South Carolina, Columbia, SC, USA

^3^Savannah River National Laboratory, Environmental Science and Biotechnology,

Aiken, SC, USA

^4^Savannah River Consulting, 301 Gateway Drive, Aiken, SC, USA

* Corresponding author: Phone: (803) 507-2714, e-mail: charles.turick@srnl.doe.gov


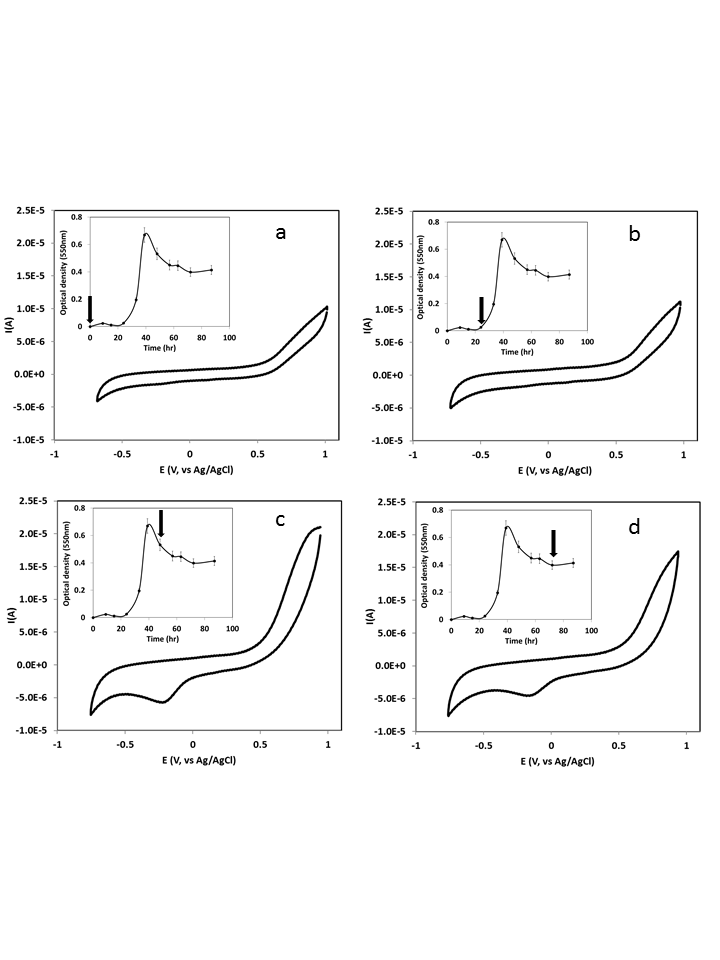


**Fig. S1** Voltammograms of *C. phytofermentans* during growth. Reduction peaks were evident in the presence of *C. phytofermentans* during growth with variations in the peak current throughout the growth cycle. Insets demonstrate the part of the growth curve that the CVs represent.


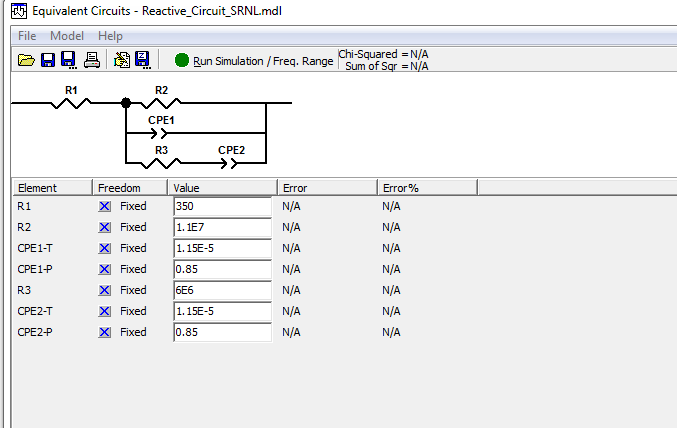

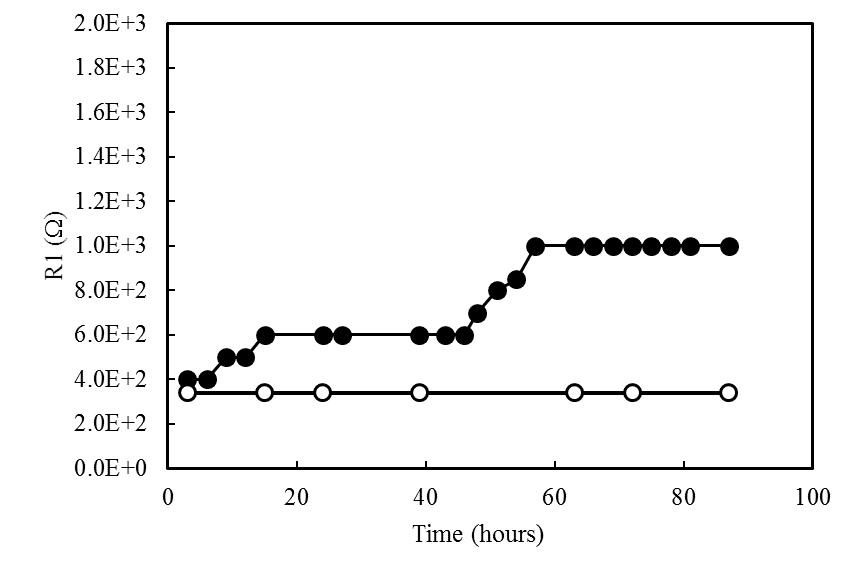


**Fig S2.** Changes in R1 during growth of *C. phytofermentans* (black circles) relative to abiotic controls (white circles).


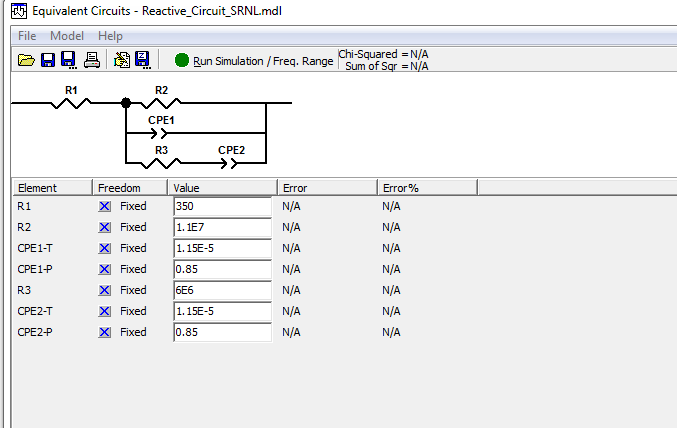

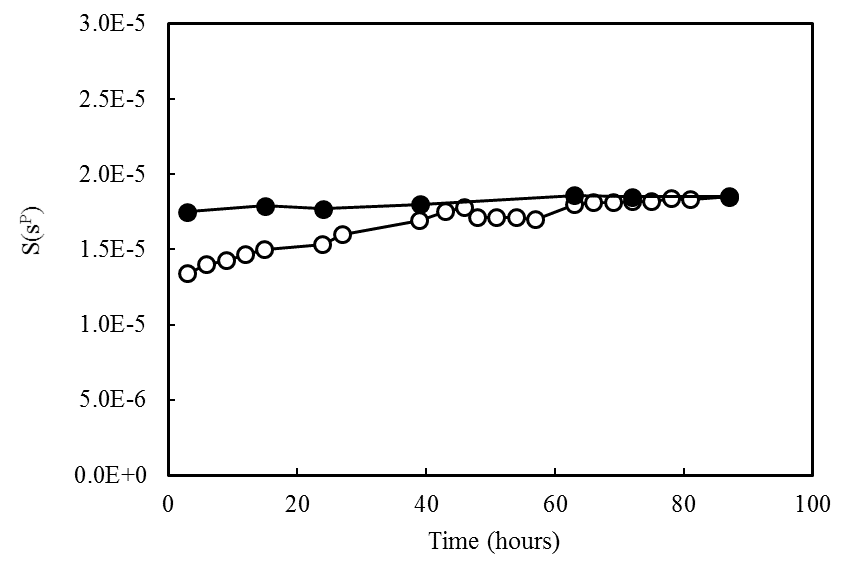


**Fig. S3**. Changes in CPE-1 and CPE-2 during growth of *C. phytofermentans* (black circles) relative to abiotic controls (white circles).
